# mTORC1 and Nutrient Homeostasis: The Central Role of the Lysosome

**DOI:** 10.3390/ijms19030818

**Published:** 2018-03-12

**Authors:** Yoana Rabanal-Ruiz, Viktor I. Korolchuk

**Affiliations:** Institute for Cell and Molecular Biosciences, Newcastle University, Newcastle upon Tyne NE4 5PL, UK

**Keywords:** mTOR, nutrient sensing, lysosome, growth, autophagy

## Abstract

The mechanistic target of rapamycin complex 1 (mTORC1) coordinates cellular growth and metabolism with environmental inputs to ensure that cells grow only under favourable conditions. When active, mTORC1 stimulates biosynthetic pathways including protein, lipid and nucleotide synthesis and inhibits cellular catabolism through repression of the autophagic pathway, thereby promoting cell growth and proliferation. The recruitment of mTORC1 to the lysosomal surface has been shown to be essential for its activation. This finding has significantly enhanced our knowledge of mTORC1 regulation and has focused the attention of the field on the lysosome as a signalling hub which coordinates several homeostatic pathways. The intriguing localisation of mTORC1 to the cellular organelle that plays a crucial role in catabolism enables mTORC1 to feedback to autophagy and lysosomal biogenesis, thus leading mTORC1 to enact precise spatial and temporal control of cell growth. This review will cover the signalling interactions which take place on the surface of lysosomes and the cross-talk which exists between mTORC1 activity and lysosomal function.

## 1. mTORC1 as a Master Regulator of Cell Growth

The mammalian target of rapamycin (mTOR) is an evolutionarily conserved serine/threonine kinase in the PI3K-related kinase (PIKK) family which coordinates cell growth and division in response to energy levels, growth signals, and nutrients [1]. It has been well established that mTOR plays the central role in the regulation of fundamental processes such as protein synthesis and autophagy, whereas deregulated mTOR signalling is implicated in diseases such as cancer, diabetes, as well as in the process of ageing [2].

mTOR nucleates two multiprotein signalling complexes, known as mTOR Complex 1 (mTORC1) and mTOR Complex 2 (mTORC2). mTORC1 consists of regulatory-associated protein of mammalian target of rapamycin (Raptor) (a scaffold protein involved in the subcellular localisation of mTORC1) [3,4], mammalian lethal with Sec13 protein 8 (mLST8, also known as GβL) (which associates with the catalytic domain of mTOR and stabilises mTOR-Raptor interaction) [3,4,5,6], and the two inhibitory subunits proline-rich Akt substrate of 40 kDa (PRAS40) [7,8,9,10] and DEP domain containing mTOR-interacting protein (DEPTOR) [11]. Although mTORC2 also encloses the core kinase protein mTOR, mLST8/GβL and DEPTOR, instead of Raptor, mTORC2 contains rapamycin-insensitive companion of mTOR (Rictor) and the regulatory subunits Sin1 [12,13,14] and Protor 1/2 [15,16]. mTORC1 and mTORC2 can be distinguished on the basis of their different sensitivity to rapamycin which upon short term application only inhibits mTORC1 [17]. The two complexes have specificity for different sets of effectors, are responsive to different signals and produce different downstream outputs. While mTORC2 regulates cytoskeletal structure, cellular metabolism, cell survival and cell response to insulin [18,19], mTORC1 predominantly regulates cellular growth by directly coordinating protein anabolism [20,21], nucleotide biosynthesis [22,23], lipogenesis [24,25,26], glycolysis [2,26,27], mitochondrial biogenesis [28], and autophagy [29,30]. mTORC1 exerts its effect through phosphorylation of key proteins such us ribosomal protein S6 kinase (S6K) and the translation repressor eukaryotic translation initiation factor 4E-binding protein (4E-BP; which is inhibited upon phosphorylation). At the same time it leads to the inhibition of autophagy by repressing lysosomal biogenesis and catabolic programs via microphthalamia-associated transcription factors (MiTF/TFE) and unc-51-like autophagy activating kinase 1 (ULK1), respectively [29,30,31,32].

Due to its role as an activator of biosynthetic and a suppressor of catabolic processes mTOR can drive cell growth and proliferation. A general assumption, very early on, was that mTOR controlled the cell cycle since loss of mTORC1 activity was shown to prevent cell cycle progression [33]. However, the proliferative role of mTORC1 was soon linked to protein synthesis at the level of translation initiation [34], suggesting that the misleading cell cycle arrest was an indirect consequence of cell growth defect and thus, uncovering the true role of mTORC1 in the control of cell growth rather than cell division. As a fundamental pro-growth pathway, mTORC1 has been unsurprisingly implicated in cellular processes in which growth is altered, such as cancer or senescence [1,35,36]. Despite seemingly opposite nature of uncontrollably proliferating cancer cells and permanently arrested senescent cells, both are characterised by the upregulation of anabolic processes [2,37]. Increased biosynthesis can either drive cell proliferation and growth of tumours or, when the cell cycle is arrested as in senescent cells, their hyperactive metabolism, protein synthesis and secretion, enlarged cell size (hypertrophy), and other senescence-associate phenotypes [36,38]. A number of reports have linked the ‘hypertrophic’ phenotype of senescence with mTORC1 activity which becomes constitutive and insensitive to external and internal mitogenic cues including growth factors and amino acids [39,40,41,42,43].

In light of this evidence, it becomes increasingly clear that such a fundamentally important signalling hub as mTORC1 requires to be tightly controlled in order to maintain healthy balance of anabolic and catabolic processes and ultimately normal physiology at the cellular, tissue, and organismal level. At the very centre of this regulation of mTORC1 signalling is the lysosome, and thereby, this organelle is now considered as a sensor and regulator of the major cellular functions in the cell [44,45,46,47].

## 2. mTORC1 Activation at the Lysosome

Lysosomes are long-recognized as the primary degradative organelle in eukaryotic cells, responsible for the breakdown of proteins, polysaccharides and complex lipids into building blocks which can then be recycled [47,48]. The distinctive degradative role of lysosomes is attributable to more than 60 different types of hydrolases which fill the lysosomal lumen and the multimeric proton pump, the vacuolar H^+^-ATPase, in the lysosomal membrane, which pumps H^+^ ions thus providing the optimal acidic environment required for the function of the luminal hydrolases [48,49]. Both intracellular and extracellular cargos can be delivered to lysosomes for degradation. Macromolecules from the extracellular space and from the cell-surface are imported to the lysosome via multiple endocytic pathways [50,51] and cytoplasmic macromolecules, damaged or misfolded proteins, and even entire organelles can be captured and delivered to the lysosome [52,53]. Hence, lysosomal catabolic processes are essential for the maintenance of metabolic homeostasis and thereby, cellular health.

The identification that mTORC1’s localisation to Rab7 and LAMP2 (lysosome-associated membrane protein 2)-positive vesicles is required for its activation [54,55] established a crucial role of the lysosomes in the regulation of mTORC1 activity and thus, in the adaptive process regulated by nutrient status and cellular signalling. The recruitment and retention of mTORC1 to the lysosome is driven by a group of small GTPases, the Rag GTPases, and a pentameric complex called Ragulator or late endosomal/lysosomal adaptor, mitogen-activated protein kinases (MAPK) and mTOR activator 1 (LAMTOR) [55]. The Rag GTPases are atypical members of the Ras superfamily of small GTPases which are stably anchored to lysosomal membranes via the resident Ragulator complex and act as docking sites for mTORC1 by directly binding Raptor [55]. Mammals have four Rag proteins that form heterodimers: Rag A or Rag B (which are highly homologous) with Rag C or Rag D (which likewise are highly similar in their sequence and functionally equivalent). The formation of heterodimeric complexes RagA/B-RagC/D is important for their stability and allows the normal activation of mTORC1 [55,56,57]. Indeed, the association between mTORC1 and Rag GTPases is highly dependent on the guanine-nucleotide binding state of the heterodimer, wherein the ‘active’ conformation consisting of RagA/B·GTP and RagC/D·GDP can bind Raptor to sequester mTORC1 to the lysosomal surface [54,55]. The unique structure of the Rag heterodimer is essential for the cross-talk between its subunits. Specifically, the binding of GTP to one subunit of the Rag heterodimer triggers a conformational change that makes it dominant over the other and drives into a locked conformation which suppresses the association of a second GTP [57]. Whereas amino acid sufficiency and glucose availability promote the accumulation of RagA/B·GTP-RagC/D·GDP, amino acid withdrawal switches the Rag heterodimer into an inactive conformation containing GDP-bound Rag A/B, thereby releasing mTORC1 from lysosomes [58]. This regulatory role of the Rags is conserved in mice [59], flies [56] and yeasts [60]. Indeed, in both mammalian and *Drosophila* cells, overexpression of GTP-bound mutants of RagA or RagB renders mTORC1 constitutively active and insensitive to amino acid withdrawal [55,56].

The study of the regulators of the nucleotide-binding status of the Rag GTPases has become of particular interest. Indeed, several nucleotide exchange factors (GEFs) and GTPase-activating proteins (GAPs) of the Rag GTPases have recently been identified. Remarkably, the Ragulator complex, besides its role in the subcellular localisation of Rag GTPases, has been shown to be responsible for their nucleotide binding state by acting as a GEF. Several GAPs have also been identified including the GAP activity towards the Rags (GATOR) 1 complex (towards RagA/B) [58], the Src-homology 3 domain-binding protein 4 (SH3BP4) towards RagB [61], the tumour suppressor folliculin (FLCN) in complex with FLCN-interacting protein 1/2 (towards RagC/D) [62] and leucyl tRNA synthetase (towards RagD) [63,64]. Furthermore, additional mechanisms to regulate the Rag GTPases have recently been unveiled, including the ubiquitination of RagA by two independent E3 ubiquitin ligases, RING-Type E3 Ubiquitin Transferase RNF152 [65] and S-Phase Kinase-Associated Protein 2 (SKP2) [66] which increases the interaction of RagA with GATOR1 and thereby, negatively regulates mTORC1 activation. This arsenal of proteins work together to ensure the right conformation of the Rag GTPases depending on the metabolic needs of the cell.

The recruitment of mTORC1 at the cytoplasmic surface of the lysosomal membrane via the Rag GTPases brings it into proximity with its activator, the small GTPase Rheb (Ras homolog enriched in brain). Rheb is a GTP-binding protein conserved from fission yeast to mammals which belongs to a unique family within the Ras superfamily of GTPases. A subpopulation of Rheb, which tethers to endomembranes through a farnesyl lipid modification on its CAAX (where C is cysteine, A aliphatic, and X terminal amino acid) motif, resides at the lysosome [55,67]. The GTP-loaded Rheb interacts with mTOR catalytic domain and robustly stimulates mTORC1 activity [68]. Whereas the regulators of GTP-loading onto Rheb remain to be established, the interaction between Rheb and mTOR is sufficient both in vitro and in vivo to promote the phosphorylation of mTORC1 substrates [69,70]. Cryo-electron microscopy structure of Rheb–mTORC1 has shed some light on the mechanism via which Rheb activates mTORC1 by showing that Rheb allosterically realigns active-site residues of mTORC1, bringing them into the correct register for catalysis [71]. An important negative regulator of Rheb is the heterotrimeric complex consisting of tuberous sclerosis complex 1 (TSC1), TSC2, and TRE-BUB2-CDC16 domain family member 7 (TBC1D7). A wide range of upstream stimuli impinges on the TSC complex. Among them, growth factor signalling (via PI3K/Akt), energy status [via the 5′ adenosine monophosphate (AMP)-activated protein kinase, AMPK], hypoxia (via the regulated in development and DNA damage responses 1, REDD) or DNA damage all of which regulate the interaction of TSC with Rheb on lysosome [72]. Hence, the TSC complex operates as an integrator of complex signalling information upstream of mTORC1. As the TSC2 subunit possesses GAP activity towards Rheb, thus converting Rheb to GDP-bound inactive form, the interaction of TSC with Rheb inhibits mTORC1 [72,73,74,75,76,77,78,79]. Additionally, binding of TSC to Rheb physically shelters it and prevents its interaction with mTORC1 [80].

Therefore, the activation of mTORC1 requires two conditions, the translocation of the complex to the lysosomal surface (a process stimulated by nutrients and Rag GTPases) where it encounters Rheb, and its activation by GTP-bound Rheb (differentially regulated by cellular signalling cascades and the availability of nutrients, energy, and oxygen). Hence, the lysosome, by providing a platform for both Rag and Rheb GTPases, creates a signalling hub that tightly controls mTORC1 activity and thereby, cellular growth, and homeostasis.

## 3. Nutrient Cross-Talk in the Regulation of mTORC1

**Figure 1:**
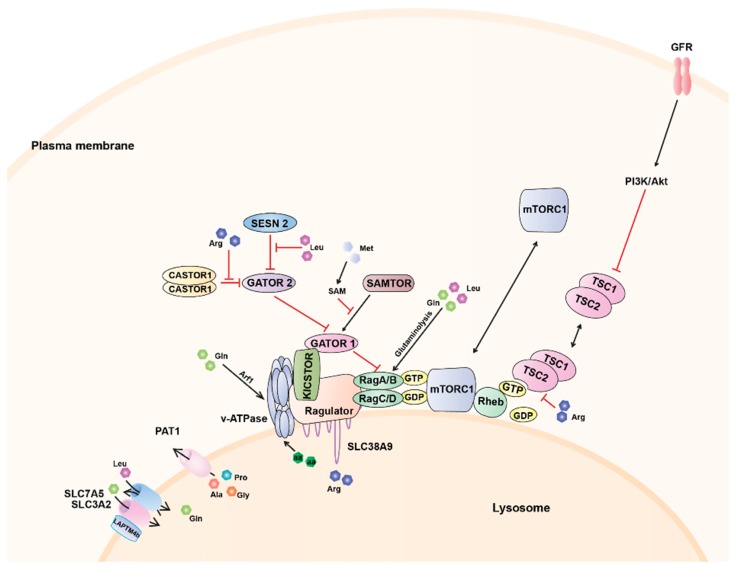
Components of the nutrient sensing pathway upstream of mTORC1. The activation of mTORC1 requires two conditions: the translocation of the complex to the lysosomal surface via Rag GTPases and the activation of Rheb GTPase. The presence of amino acids is essential for the activation of mTORC1 as they control the nucleotide-loading status of the Rag GTPases. Amino acids signal to mTORC1 by different mechanisms including cytoplasmic sensors (SESN2, CASTOR1, and SAMTOR), amino acid transporters (SLC38A9, PAT1, or SLC7A5/SLC3A2) and the v-ATPase on the lysosomal membrane. Growth factors (including hormones, cytokines, and chemokines) activate receptor tyrosine kinases or G-protein-coupled receptors, which, through various mechanisms, activate PI3K/Akt. When active, Akt phosphorylates and inhibits the TSC complex thereby relieving the inhibition of Rheb and allowing it to become activated and stimulate mTORC1 kinase activity. Arrows indicate stimulation and blocked arrows indicate inhibition. GFR, growth factor receptor; SESN, Sestrin; GATOR; TSC, tuberous sclerosis complex; GTP, guanosine-5′-triphosphate; GDP, guanosine-5′-diphosphate; PAT, proton-assisted amino acid transporter; SLC, solute carrier family; SAM, S-adenosylmethionine; CASTOR cytoplasmic arginine sensor for mTORC1; SAMTOR, S-adenosylmethionine sensor for mTORC1.

As described above, at the lysosome, amino acids, and growth factors cooperate to ensure proper mTORC1 activation by controlling its colocalisation with Rheb and Rheb activation, respectively (Figure 1). Specifically, amino acid sufficiency promotes the recruitment of mTORC1 to the lysosomal surface via its interaction with the Rag GTPases [54] bringing mTORC1 into close proximity with the lysosomally localised Rheb. Upon growth factors scarcity, the TSC complex is accumulated at the lysosome inhibiting Rheb activation. However, in response to growth factor-stimulated PI3K signalling, Akt phosphorylates TSC2 inducing its dissociation from the lysosome [79,81] and ultimately allowing mTORC1 activation.

Hence, amino acid-induced mTORC1 tethering to the lysosomal membrane is fundamental in the modulation of Rheb/TSC-mediated mTORC1 activation, thus providing a mechanism that would explain why activation of mTORC1 by growth factors requires the presence of amino acids. Furthermore, although the TSC2/Rheb signalling input has classically been considered to be insensitive to amino acids [68,82,83], the regulation of TSC2 localisation to the lysosome has recently been associated with the amino acid/Rag GTPase axis [84]. Specifically, in the absence of amino acids, TSC2 is lysosomally localised, even in the presence of growth factor signalling. Both amino acids and growth factor signalling are required to maintain TSC2 cytoplasmic; when one of the two is missing, TSC2 relocalises to lysosomes [72,84]. Recent studies have demonstrated the ability of specific amino acids to regulate the localisation of the TSC complex. In particular, arginine has been shown to suppresses lysosomal localisation of the TSC complex and its interaction with Rheb. By interfering with TSC-Rheb complex, arginine relieves allosteric inhibition of Rheb by TSC [80]. Thus, the underlying mechanisms controlling the opposing localization of mTORC1 and TSC to the lysosome involve complex interplay between different upstream stimuli. Growth factors signalling via PI3K/Akt/Rheb can also potentiate amino acid-dependent mTORC1 activation by regulating the lysosomal localisation of the proton-assisted amino acid transporter 1 (PAT1), a member of the PAT or SLC36 family and an essential mediator of amino acid-dependent mTORC1 activation (described below) [85]. These findings highlight the cooperation of different nutrient inputs in the activation of mTORC1 on lysosomes which integrate internal and external cues to regulate cell metabolism, growth, proliferation and ultimately, survival.

### Amino Acid-Dependent Regulation of mTORC1 

Increasing evidence suggests that amino acids are able to signal to mTORC1 through different mechanisms from inside and outside the lysosome, via both, the cytoplasm and the lysosomal lumen. The characterization of the interaction between the Ragulator complex and the lysosomal proton pump, the v-ATPase, led to the identification of the v-ATPase as a new component of the nutrient sensing machinery in the lysosome and revealed that lysosomal-lumen amino acids can be sensed by mTORC1 [86]. An increase in the intra-lysosomal amino acid concentration promotes a conformational change in the v-ATPase that weakens its binding with the Ragulator complex [86]. These observations led to propose a mechanism of amino acid sensing where the v-ATPase would act as a sensor [86]. This lysosome-centric model called “inside-out” mechanism proposes that amino acids must accumulate in the lysosomal lumen and signal through the v-ATPase to the Ragulator and ultimately, the Rag GTPases [86]. Open questions, that remain unanswered, include the precise mechanism by which the v-ATPase senses amino acids, and specifically, the role of the ATPase activity for mTORC1 activation. Whereas pharmacological inhibition of its ATP hydrolysis activity led to mTORC1 inhibition [86,87], others have shown an increase in the ATPase activity after amino acid starvation [88]. Amino acid deprivation appears to promote conformational changes in the v-ATPase [86] which could increase its assembly [88] and its interaction with the Rag GTPases-Ragulator complex. Furthermore, it has been demonstrated that intraluminal pH of the lysosome in osteoclasts is important to support mTORC1 activity [89].

It still remains to be elucidated whether the sensing of amino acids in the lysosomal lumen involves amino acids liberated via the degradative nature of the lysosome or amino acids trafficked from the cytoplasm into the lysosome. Stimulation of starved cells with radiocarbon (14C)-labelled amino acids leads to the rapid accumulation of these amino acids in isolated lysosomes [86]. Furthermore, their transport and accumulation within the lysosomal lumen does not appear to be driven by a proton gradient, as they enter the lysosome regardless of the v-ATPase activity [86,90]. How amino acids are transported into the lysosome and what particular transporters would facilitate this process is still unknown.

In contrast, the efflux from the lysosome of most essential amino acids has been shown to be regulated by a process which involves mTORC1 itself and SLC38A9, a sodium-coupled amino acid transporter which directly interacts with the Ragulator-Rag GTPase complex [91,92]. SLC38A9 constitutes the main transporter that has been firmly implicated in sensing of specific amino acids from within the lysosome [91,92]. Despite its higher affinity for amino acids such us glutamine [93], SLC38A9 participates in arginine sensing following starvation and refeeding [91,94]. Overexpression of this transporter caused sustained mTORC1 activation whereas mTORC1 activation was abolished in SLC38A9 knockout cells [95]. Furthermore, knockdown of SLC38A9 impairs starvation-induced relocalisation of mTOR to the cytoplasm [95] though the mechanism of mTOR retention is still unknown. A recent study has reported that SLC38A9 is required for the efflux of essential amino acids from the lysosome in an arginine-regulated fashion [94]. They have also demonstrated that SLC38A9 is necessary for the exit of leucine from the lysosomes and the activation of mTORC1 and proposed SLC38A9 as a new player in the regulation of amino acid homeostasis by acting as a lysosomal messenger that couples mTORC1 activation to the release of essential amino acids to drive cell growth [94].

Other lysosomal amino acid transporters have also been shown to modulate activity of mTORC1. The heterodimeric transporter SLC7A5 (LAT1)/SLC3A2 can be recruited to the lysosomal membrane via a protein called LAPTM4b [96], wherein it stimulates mTORC1 activation via the v-ATPase by coupling the import of leucine with the simultaneous efflux of glutamine [97]. Likewise, as mentioned above, the amino acid transporter PAT1, has been shown to be concentrated at the surface of late endosomes and lysosomes in many cell types, wherein it interacts with the Rag GTPases [85,98]. This amino acid transporter is involved in the transport of alanine, glycine and proline [99,100] and it has been suggested to mediate the regulation of amino acid-dependent mTORC1 localisation [85]. Overexpression of PAT1 inhibits mTORC1 activation [86], likely due to the leak of amino acids from the lysosome. However, other studies have suggested that PAT1 overexpression can either promote growth and mTORC1 signalling in vivo and in cell culture [98,101] or inhibit these processes when expression levels are raised further, potentially via a dominant negative mechanism [86,101]. The localisation of PAT1 in the lysosomal surface has recently been shown to be regulated by FLCN levels. Specifically, FLCN overexpression leads to decrease in PAT1 localisation to the lysosome, thus sequestering amino acids such us leucine within the lysosome maintaining the activation of mTORC1 even in amino acid-limited conditions [102]. Intriguingly, FLCN is normally recruited to the lysosome via Rags upon amino acid starvation. Nevertheless, endogenous levels of FLCN could not be sufficient to affect PAT1 and intracellular amino acid levels, and thus, mTORC1 is switched off [103].

The abovementioned lysosomal amino acid transporters provide a logical explanation that amino acids exported from the lysosome to the cytoplasm would be sensed by mTORC1. However, future work is likely to unveil the precise mechanism via which these transporters contribute to control intracellular concentrations of amino acids and subsequently, mTORC1. Increasing evidences exist suggesting that the role of amino acid transporters in amino acid sensing is not only due to their ability to translocate amino acids through the lipid bilayer, but also by acting as activators of amino-acid dependent signalling either in the presence or the absence of transport. This dual receptor-transporter function for the amino acid transporters would let them operate as ‘transceptors’ by sensing amino acid availability upstream of intracellular signalling pathways [104,105]. This new concept may help to understand why SLC38A9 senses arginine despite its affinity for glutamine [93], or the influence of PAT1 on the cellular growth when it transports amino acids that are not potent activators of mTORC1 [99,100].

The influx/efflux and/or accumulation of amino acids into the lysosomal lumen are somehow detected by the lysosomal molecular machinery and future work is likely to increase our understanding of how this transport is orchestrated. A plausible explanation is that both, the v-ATPase and the lysosomal transporters cooperate to ensure a fine control of the amino acids sensing at the lysosome. According to this idea, v-ATPase inhibition in cells overexpressing SLC38A9 does not inhibit mTORC1 signalling upon amino acid withdrawal [93]. Furthermore, it has been suggested that the PAT/Rag/Ragulator/v-ATPase complex is required to establish a microenvironment for cycling protons and export amino acids that is needed to drive the amino acid sensing at the lysosome [85]. This coupling of the v-ATPase and PATs may explain why the reduction in the proton gradient across the lysosomal surface does not block amino acid-dependent mTOR relocalisation [85,86].

The lysosomal lumen environment, therefore, provides a complex readout of amino acid levels for mTORC1. Nevertheless, some of the major contributors to mTORC1 activation are also sensed outside the lysosome [64,106,107,108,109,110]. Indeed, specific cytoplasmic sensors for leucine and arginine have been recently identified [106,111,112,113,114,115]. Leucine and arginine signal mTORC1 through a distinct pathway comprising the GATOR1 and GATOR2 complexes [116]. GATOR1 contains three proteins, DEP domain containing 5 (DEPDC5), nitrogen permease regulator 2-like protein (NPRL2), and NPRL3. GATOR1 is tethered to the lysosomal surface through the multiprotein complex KICSTOR [117] wherein it interacts directly with RagA/B acting as a GAP thus leading to mTORC1 inhibition [58,118]. GATOR2 is composed of five components, meiosis regulator for oocyte development (MIOS), WD repeat domain 24 (WDR24), WD repeat domain 59 (WDR59), SEH1 like nucleoporin (SEH1L), and SEC13 and this complex negatively regulates GATOR1 thereby relieving mTORC1 from its inhibition [58,118]. The inhibitory effect of GATOR2 on GATOR1 is mediated by Sestrin (SESN) 2 [111,119]. Specifically, SESN2 binds and inhibits GATOR2 function in the absence of leucine, whereas leucine binding dissociates it from GATOR2 [114,115]. Furthermore, SESN2 has recently been proposed an indirect mediator of prolonged amino acid starvation as its expression is induced by the stress-responsive activating transcription factor 4 (ATF4) upon long-term starvation [120].

Besides its role in the regulation of the TSC complex localisation described above, and via a mechanism similar to that of leucine, arginine binding to CASTOR1 (cellular arginine sensor for mTORC1) disrupts the interaction CASTOR1-GATOR2 which enables GATOR2 to inactivate GATOR1, thereby leading to increased Rag-dependent mTORC1 signalling [106,112].

More recently, SAMTOR, a sensor for methionine, has been identified [121]. Unlike leucine and arginine, which directly bind sensors upstream of mTORC1, methionine is sensed indirectly through S-adenosylmethionine. SAMTOR potentiates GATOR1 function via an unknown mechanism that may involve the disruption of the binding GATOR1-GATOR2. The interaction between SAMTOR and GATOR1 also requires KICSTOR, which could reflect either a composite binding site or the requirement of KICSTOR to localise GATOR1 to the lysosomal surface. The binding of S-adenosylmethionine to SAMTOR disrupts its interaction with both GATOR1 and KICSTOR [121] and activates mTORC1 signalling.

Glutamine also activates mTORC1 by a Rag GTPase-dependent mechanism. Specifically, the production of α-ketoglutarate (α-KG) in the mitochondria via glutaminolysis stimulates GTP loading of RagB [107,108]. This metabolic process is supported by leucine availability providing a mechanistic link between leucine and glutamine in glutaminolysis-dependent regulation of mTORC1 activation. However, glutamine can also activate mTORC1 independently of the Rag GTPases in a mechanism dependent on the v-ATPase and the adenosine diphosphate ribosylation factor-1 (Arf1), a key regulator of intracellular vesicle trafficking [110].

## 4. Other Inputs in the Regulation of mTORC1 

Whereas mTORC1 is generally activated by growth- and proliferation-stimulating signals, its activity can be reduced and even suppressed when cells are exposed to a variety of stress conditions. A substantial part of mTORC1 inhibitory inputs, such us intracellular energy levels, cytokines, or hypoxia, are channelled through the TSC complex [122]. The regulation of mTORC1 by energy status appears primarily mediated through AMPK, which functions as a heterotrimeric protein consisting of a catalytic α-subunit, a β-subunit and an adenosyl nucleotide-binding γ-subunit [123]. This complex is sensitive to changes in the cellular AMP/ATP ratio and AMP binding allosterically activates it. Once active, AMPK, through phosphorylation of TSC2, promotes mTORC1 inhibition and thus catabolic pathways (i.e., autophagy) in order to recover cellular energy homeostasis and attenuates anabolic pathways to preserve energy (described below) [44,123]. Furthermore, a decrease in cellular ATP levels can result in impaired protein folding and ER stress, as ATP synthesis is required for chaperone activity in the ER [124]. The lack of activity of ER chaperones generate an imbalance between protein synthesis and protein folding capacity which leads to accumulation of unfolded proteins in the ER lumen and results in ER stress [125,126]. Under these conditions, mTORC1 is inactivated and autophagy activation is triggered as a protective response to the overload of unfolded or misfolded proteins that exceed the capacity of the proteasome. ER stress limits mTORC1 activity by stimulating the TSC complex [127].

The adaptive response to hypoxia, low oxygen levels, also involves inhibition of mTORC1 in a TSC2-dependent manner. An essential component for hypoxia regulation of mTORC1 activity is the stress-induced protein REDD1 [128], which is also upregulated through transcriptional mechanisms in response to DNA damage, ER stress, serum deprivation, glucocorticoid-hydrogen peroxide or dexamethasone-treatment [129,130,131,132,133]. Together, all these mTORC1-suppressive mechanisms settle the careful balance of mTORC1 activation required for the maintenance of cellular homeostasis.

## 5. Cross-Talk between mTORC1 and Lysosomes

**Figure 2:**
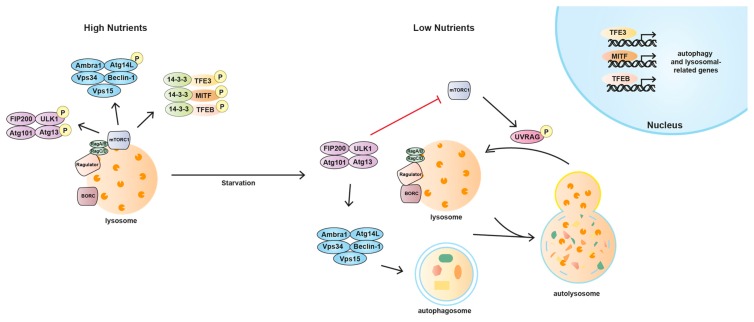
mTORC1 and the lysosome manage between anabolic and catabolic processes. Under nutrient-rich conditions, mTORC1 phosphorylates and inhibits the autophagy initiators ULK1 and Atg13. mTORC1 also phosphorylates the transcription factors MITF, TFEB, and TFE3 which facilitates the interaction of these receptors with the cytosolic chaperone 14-3-3 and thus, retains them in the cytosol. Conversely, in the absence of nutrients, mTORC1 relieves the inhibition of these proteins leading to the transcription of lysosomal and autophagy-related genes and the induction of autophagy. Arrows indicate stimulation and blocked arrows indicate inhibition. TFE3, transcription factor E3; MITF, microphthalmia-associated transcription factor; TFEB, transcription factor EB; BORC, BLOC-one-related complex; UVRAG, UV irradiation resistance-associated gene.

Lysosomes are not mere platforms for proper assembly of the mTORC1 regulatory pathway. mTORC1 plays a crucial role in the lysosomal function by regulating biogenesis, distribution, and activity of lysosomes [31,44,46,47]. Thus, the liaison between mTORC1 and lysosomes provides this organelle with the ability to adapt to environmental cues by sensing its nutrient content and generating a response which controls cellular clearance by autophagy, energy metabolism, and ultimately growth and survival [46] (Figure 2).

### 5.1. Lysosomal Biogenesis

Cells constantly monitor lysosomal function and possess the ability to upregulate the number and activity of the lysosomes in response to the increase in their energetic needs. The four members of the MiTF/TFE family in mammals, microphthalmia-associated transcription factor (MITF), transcription factor EB (TFEB), transcription factor 3 (TFE3), and transcription factor EC (TFEC), are basic helix-loop-helix leucine zipper transcription factors that control basic cellular processes in eukaryotes as well as tissue identity and differentiation in animal development [134,135]. Recent studies in mammalian cell lines have implicated MITF, TFEB, and TFE3 in the regulation of lysosomal biogenesis and degradation pathways [136,137,138,139,140,141]. Expression profiling has shown that these transcription factors induce the biogenesis of lysosomes and autophagosomes and the clearance of cellular debris under conditions of starvation or lysosomal stress [135,136,137,138,142,143,144]. The activity of MITF, TFEB, and TFE3 is dictated by their nuclear localisation, which is regulated by mTORC1 and nutrient levels [141]. Phosphorylation of these transcription factors by mTORC1 requires their recruitment to lysosomes, wherein active mTORC1 resides. This tethering is mediated by the direct interaction of an N-terminal motif present in all three MiTF members with the Rag GTPases [145]. Then, phosphorylation of the transcription factors by mTORC1 in critical serine residues mediates their interaction with the cytosolic chaperone 14-3-3 and causes their retention in the cytosol [136,141,146]. These phosphorylated residues have been characterised as Ser211, Ser321, and Ser173 in TFEB, TFE3, and MITF, respectively [31,146,147,148].

Following starvation, mTORC1 inactivation leads to the dissociation of the transcription factor/14-3-3 complex which allows their transport to the nucleus and the expression of lysosomal and autophagy related genes, including proteins implicated in the formation of autophagosomes, autophagosome-lysosome fusion, and cellular clearance [140,149]. Interestingly, a recent work has demonstrated the presence of a feedback loop by which TFEB, TFE3, and MITF can influence mTORC1 activity by regulating the expression of RagD [150]. The fine modulation of this transcriptional regulatory mechanism is critical for cellular adaptation to nutrient availability while its deregulation supports cancer metabolism, thus promoting tumour growth [150]. TFEB is considered the master regulator of the lysosomal biogenesis since it binds to the Coordinated Lysosomal Expression and Regulation (CLEAR) element, enriched in the promoter regions of numerous lysosomal genes, thereby promoting their transcription [136,137,139,142,143]. Interestingly, both TFEB and TFE3 induce the expression of FLCN [147], suggesting the presence of a regulatory loop in which TFEB (and TFE3) might contribute to mTORC1 reactivation and therefore their own inhibition. TFE3, besides its role in the biogenesis of lysosomes and autophagosomes and the clearance of cellular debris, has also been recently described to play a role in the maintenance of stem cell pluripotency [151]. Re-localisation of TFE3 from the nucleus to the cytosol was shown to be required for embryonic stem cells to exit their native pluripotent state and commit to cellular differentiation. This cytosolic retention of TFE3 is controlled by the activity of the tumour suppressor FLCN, its binding partners FNIP1/2 and the mTORC1 regulators TSC1 and TSC2 [151,152]. MITF, when freed from mTORC1-induced sequestration, drives transcription of all the subunits of the v-ATPase. A recent study has reported that, through the v-ATPase, MITF feeds back onto mTORC1 which in turn, negatively regulates MITF [153]. This MITF/v-ATPase/mTORC1 regulatory loop adjusts the activity of all three players offering a mechanism for continuously balancing metabolic pathways as the nutritional state of the cell fluctuates. In this model, the level of v-ATPase would sensitize or desensitize the nutritional sensing mechanism to changes in amino acid levels [153]. Such mechanism would impose a limit on upregulation of catabolism under low nutrient conditions, and an upper limit on active TORC1 and its promotion of anabolic pathways when nutrients are abundant, thus providing a feedback mechanism to maintain cellular homeostasis.

Finally, the transcription factor zinc finger with KRAB and SCAN domains 3 (ZKSCAN3), a zinc-finger family DNA-binding protein, which is considered a master repressor of lysosomal biogenesis and autophagy [154], is also directly regulated by mTORC1. Under nutrient rich conditions ZKSCAN3, by virtue of repressing the expression of essential genes for multiple autophagy steps, keeps this cellular process in check. However, under sustained starvation conditions, ZKSCAN3 is translocated from the nucleus thereby de-repressing this network of genes, allowing for an activated autophagy response [154]. Thus, ZKSCAN3 and TFEB are oppositely regulated by starvation and in turn oppositely regulate lysosomal biogenesis and autophagy, suggesting that they act in conjunction. Supporting this hypothesis, parallel ZKSCAN3 repression and TFEB upregulation has an additive effect on autophagy and lysosome biogenesis [154].

Overall, the close collaboration between mTORC1 and other master regulators of lysosomal biogenesis and autophagy creates a homeostatic loop that regulates the activity of each of these factors and facilitate an efficient response to the varying nutrient demands of the cell [31].

### 5.2. Lysosomal Function: Autophagy 

Autophagy is an evolutionarily conserved and tightly regulated lysosomal pathway that degrades cytoplasmic materials and organelles [53,155]. In the predominant form of autophagy called macroautophagy, this multistep degradation process includes the induction of autophagosomes, fusion of autophagosomes to lysosomes to generate autolysosomes and fission of autolysosomes to release lysosomes and to terminate autophagy [53]. All these mechanistically distinct steps are tightly regulated by the coordination of mTORC1 activity and the core autophagic machinery.

Autophagy is initiated through the ULK1 complex. This complex consists of the core Ser/Thr kinase ULK1 itself, the scaffold protein focal adhesion kinase family interacting protein of 200 kDa (FIP200) and the HORMA (Hop/Rev7/Mad2) domain-containing proteins Atg13 and Atg101 [29,30,156,157,158,159,160,161]. ULK1 is regulated by amino acids and energy status via mTORC1 and AMPK, respectively. When mTORC1 is active, it phosphorylates both ULK1 and Atg13, which reduces ULK1 kinase activity [29,30,159,162,163]. However, under starvation conditions, when mTORC1 is inhibited, it dissociates and relieves the inhibition of ULK1 [30]. AMPK responses to low energy levels inactivating mTORC1 and, thereby, indirectly activating the ULK1 complex. However, AMPK also directly phosphorylates ULK1 and Atg13 to stimulate autophagy in most of the cases, although one study has shown inhibitory effect [162,164,165,166]. At the same time, the ULK1 complex negatively regulates the kinase activity of mTORC1 by phosphorylating Raptor and reducing its ability to bind the substrate eukaryotic translation initiation factor 4E-binding protein (4E-BP1). This negative feedback loop occurs upon activation of autophagy to maintain mTORC1 inhibition when nutrient supplies are limiting and hence may be important to coordinately regulate cell growth and autophagy with optimised utilization of cellular energy [167,168]. Downstream the ULK1 complex, the Vps34 complex it is also required for autophagy induction. This complex includes Atg14, Vps15, and Beclin-1. When nutrients are plentiful, mTORC1 can phosphorylate Atg14 in order to inhibit the autophagosome biogenesis and down-regulate autophagy. Conversely, upon nutrient withdrawal mTORC1 inhibition relieves this pro-autophagy complex to stimulate autophagy [169]. Additionally, mTORC1 is involved in the fusion between autophagosomes and lysosomes, which seems to require the inactivation of the complex [170]. It has been reported that the synthetic compound MHY1485 inhibits lysosomal fusion during starvation-induced autophagy by directly binding and activating mTORC1 [170].

During the initial steps of autophagy mTORC1 is downregulated, though it becomes reactivated several hours later, with its reactivation dependent on the release of intracellular nutrients during the autophagic process [149]. This increased mTORC1 activity inhibits further autophagy and permits the generation of proto-lysosomal tubules and vesicles which extend from autolysosomes and ultimately mature into functional lysosomes, thereby restoring the full complement of lysosomes in the cell [149]. Lysosomal regeneration is dependent on the phosphorylation of the UV irradiation resistance-associated gene (UVRAG) by mTORC1. When phosphorylated by mTORC1, UVRAG enhances Vps34 lipid kinase activity which appears to be critical for tubule scission [171]. This feedback mechanism tightly couples nutritional status to the induction and termination of autophagy.

It is widely accepted that the localization of mTORC1 in lysosomes led this complex to regulate lysosomal metabolism and autophagy. However, the lysosome itself can also feedback to mTORC1 signalling. Besides the generation of amino acids during autophagy which permits the reactivation of mTORC1 as previously described [149], it has been reported that, the pharmacological inhibition of lysosomal function in chondrocytes using Concanamycin A leads to the activation of mTORC1 in both autophagy- and nutrient-independent manner [172]. This cross-talk also operates in reverse, where inappropriate mTORC1 activity prevents lysosomal activation under starvation [173]. Another example in the coupling of lysosomal function and mTORC1 activity is the autophagy receptor p62 (also known as sequestosome 1, SQSTM1). Besides its well-known role as a mediator of selective autophagy of ubiquitinated proteins [174,175], p62 interacts with the mTORC1 complex component Raptor and the Rag GTPases [176], thus providing a signalling nexus for mTORC1 activation at the lysosome. Specifically, p62 favours the formation of the active Rag heterodimer and, together with the tumor necrosis factor (TNF) receptor associated factor 6 (TRAF6) promotes mTORC1 translocation to the lysosome [177].

All these observations have shed some light on the integration of both anabolic and catabolic processes in the lysosome and especially the tight regulation of both processes by a coordination of mTORC1 and lysosomal activity. Although beyond the main scope of this review, it is also important to mention that autophagy can be modulated in an mTORC1-independent manner. For example, mTORC1-independent regulation of autophagy by the inositol signalling pathway has been identified where an elevation of intracellular inositol inhibits autophagosome synthesis [178]. Furthermore, inositol-lowering agents, such us lithium, carbamazepine, or valproic acid have been shown to induce autophagy and facilitate the clearance of autophagy substrates without inhibiting mTORC1 activity [179,180].

### 5.3. Lysosomal Positioning

In addition to controlling the localisation of mTORC1, nutrients, and mitogenic signals also regulate the subcellular distribution of lysosomes [181]. Under nutrient-replete conditions, lysosomes tend to distribute closer to the plasma membrane, whereas starvation leads to the retrograde transport and clustering of lysosomes in the perinuclear area of the cell [181]. The multiple functions of lysosomes are dependent on their ability to undergo bidirectional movement between the centre and the periphery of the cell, and it might be possible that nutrient-dependent changes in lysosomal membrane potential or mTORC1 activity regulate the association of lysosomes with microtubules or specific motors [182].

A plethora of proteins have been shown to regulate the lysosomal positioning, including the recently reported multisubunit complex BORC (BLOC1-related complex) [182,183], which associates with the cytosolic face of the lysosomal membrane wherein recruits the Arf-like small GTPase Arl8b from the cytosol to the lysosomal membrane [183], and ultimately promotes the peripheral movement of lysosomes [183]. It has been described that BORC can be directly regulated by the Ragulator, which binds BORC upon amino acid starvation and negatively regulates its function in the movement of lysosomes toward the cell periphery. This mechanism provides a logical explanation for the juxtanuclear clustering of lysosomes induced by nutrient depletion. Interestingly, the effect of Ragulator on BORC appears to be independent of mTORC1 signalling [184].

In addition, both the phosphatidylinositol 3,5-biphosphate-transient receptor potential cation channel, mucolipid subfamily-asparagine-linked glycosylation 2 (PI(3,5)P2-TRPML-ALG-2) pathway [185] and the FLCN-Rab interacting lysosomal protein (RILP)-Rab4 complex [186] are required for the centripetal movement of lysosomes towards the perinuclear region upon autophagy induction. Specifically, it has been described that maintenance of perinuclear lysosomes may be mediated by the starvation-induced FLCN association with lysosomes which recruits RILP and drives the formation of contact sites between lysosomes and the Golgi-associated small GTPase Rab34 which ultimately restricts lysosome motility and thus, promotes their retention in this region of the cell [186]. Intriguingly, the transient receptor potential cation channel, mucolipin subfamily (TRPML) has been reported to be inactivated by mTOR through phosphorylation which suggests a novel mechanism in the regulation of the perinuclear retention of lysosomes [187].

Genetic manipulation of the distribution of lysosomes has been shown to directly impinge on mTORC1 activity. Indeed, localisation of lysosomes at the cell periphery correlates with increased mTORC1 activity whereas the inhibition of lysosomal scattering results in diminished mTORC1 activity and, consequently, an increase in the number of autophagosomes [181]. However, how lysosomal positioning has an impact on mTORC1 activity is currently unknown. The closer localisation of lysosomes to the cell periphery in the presence of nutrients has been suggested to promote the activation of mTORC1 via signalling cascades originating from the plasma membrane [181], although the mechanisms controlling the sequestration of lysosomes in these regions are not known. In adipocytes, insulin and amino acid-dependent increase in PI(3,5)P2 recruits mTORC1 to the plasma membrane via Raptor [188]. It would be interesting to elucidate whether this interaction could also control mTORC1-positive lysosome localisation. On the other hand, clustering of lysosomes in the perinuclear area upon nutrient withdrawal may facilitate encounter of lysosomes with autophagosomes, promoting fusion of these organelles and maintenance of autophagic flux [181]. A recent study has proposed that the local PI(3,4)P2 synthesis by the class II PI3Kβ (PI3KC2β) at the lysosome triggers recruitment of inhibitory 14-3-3 proteins to locally repress mTORC1 signalling and to reposition lysosomes at the cell centre when growth factor signalling ceases. However, the mechanism via which PI3KC2β senses the absence of growth factors remains to be determined [189]. Future studies will have to address how all of those processes are coordinated in the cell to tightly control spatiotemporal changes in lysosome positioning in response to nutrient availability.

## 6. Concluding Remarks

The ability of cells to respond appropriately to fluctuations of nutrient availability is fundamental for their adaptation to the environment. Cells have developed sophisticated mechanisms to adapt their metabolism to different levels of nutrients. Depending on the conditions of nutrient availability or metabolic stress cells modulate the rate of anabolism and catabolism accordingly. In both processes, the master regulatory complex mTORC1 is a central player which acts as a signal integrator by driving cell growth and proliferation through the activation of protein, pyrimidine, and lipid biosynthesis when conditions are favourable and, at the same time, suppressing autophagy, the major catabolic pathway [190,191,192]. Given all the signals that impinge on mTORC1, it is not surprising that it requires a plethora of molecular accessory factors controlling its activity. Rag and Rheb GTPases constitute two parts of a mechanism that ensures that mTORC1 becomes activated only when nutrients and growth factor conditions are both appropriate [193]. In the current model, the Rag GTPases are essential for amino acid signalling to mTORC1, recruiting mTORC1 to the lysosome in response to amino acids so that Rheb can stimulate its kinase activity if growth factors (and energy) are available. Over the last few years there have been reported a variety of pathways by which amino acids can trigger mTORC1 activation [190,192,194]. These mechanisms include cytoplasmic sensors (SESN2, CASTOR1, and SAMTOR), amino acid transporters (SLC38A9, PAT1, or SLC7A5/SLC3A2) and the v-ATPase on the lysosomal membrane, which are covered in more detail elsewhere [190]. Growth factors, however, signal via the PI3K/Akt signalling cascade to phosphorylate and inhibit the TSC complex, relieving the inhibition of Rheb and allowing it to become activated and stimulate mTORC1 kinase activity. Amino acids and growth factors collaborate much more closely in regulating mTORC1 than previously thought. Indeed, the regulation of TSC2, which has been classically considered to be sensitive to growth factors, has recently been associated with amino acids [80,84], and the trafficking of amino acid transporters to the lysosome has been shown to be regulated by growth factors [85]. These findings, in addition to reinforce the cooperation of different nutrient inputs in mTORC1 activation, highlight the integration of growth factor and amino acid-dependent signals upstream mTORC1 at the lysosome.

The identification of lysosomes as scaffolding platforms on which mTORC1 becomes activated has had a profound impact on the view of these organelles which have been traditionally considered static organelles, with no influence from the external environment. The lysosome is now gaining prominence as a nutrient signalling hub which integrates internal and external cues, with the v-ATPase playing an important role in sensing amino acid availability, and in signalling pathways that are involved in cell metabolism and growth [31,46,47,195]. The ability of the lysosome to adapt their metabolism to different environment allows extremely tight spatial coupling between autophagy and cellular growth and proliferation. Understanding the functional activation of the lysosome and the underlying mechanisms, especially the regulatory effect of mTORC1, would help to elucidate the integration of anabolic and catabolic processes in the cell and expand the scope of mTORC1 function in the regulation of autophagy and cell growth.
